# Efficacy and safety of high-flow nasal cannula oxygen therapy in moderate acute hypercapnic respiratory failure

**DOI:** 10.5935/0103-507X.20190026

**Published:** 2019

**Authors:** Maria Eugenia Yuste, Olga Moreno, Susana Narbona, Fernando Acosta, Luis Peñas, Manuel Colmenero

**Affiliations:** 1 Unidade de Terapia Intensiva, Hospital Universitário San Cecilio - Granada, Espanha.; 2 Instituto de Investigación Biosanitaria - Granada, Espanha.

**Keywords:** Respiratory insufficiency/therapy, Oxygen inhalation therapy/methods, Oxygen/therapeutic use, Cannula/utilization, Respiration, artificial, Intensive care units, Insuficiência respiratória/terapia, Oxigenoterapia/métodos, Oxigênio/uso terapêutico, Cânula/utilização, Respiração artificial, Unidades de terapia intensiva

## Abstract

**Objective:**

To assess the efficacy and safety of high-flow nasal cannula oxygen therapy in treating moderate hypercapnic respiratory failure in patients who cannot tolerate or have contraindications to noninvasive mechanical ventilation.

**Methods:**

A prospective observational 13-month study involving subjects admitted to an intensive care unit with or developing moderate hypercapnic respiratory failure. Clinical and gas exchange parameters were recorded at regular intervals during the first 24 hours. The endpoints were a oxygen saturation between 88 and 92% along with a reduction in breathing effort (respiratory rate) and pH normalization (≥ 7.35). Subjects were considered nonresponders if they required ventilatory support.

**Results:**

Thirty subjects were treated with high-flow nasal cannula oxygen therapy. They consisted of a mixed population with chronic obstructive pulmonary disease exacerbation, acute cardiogenic pulmonary edema, and postoperative and postextubation respiratory failure. A nonsignificant improvement was observed in respiratory rate (28.0 ± 0.9 *versus* 24.3 ± 1.5, p = 0.22), which was apparent in the first four hours of treatment. The pH improved, although normal levels were only reached after 24 hours on high-flow nasal cannula therapy (7.28 ± 0.02 *versus* 7.37 ± 0.01, p = 0.02). The rate of nonresponders was 13.3% (4 subjects), of whom one needed and accepted noninvasive mechanical ventilation and three required intubation. Intensive care unit mortality was 3.3% (1 subject), and a patient died after discharge to the ward (hospital mortality of 6.6%).

**Conclusion:**

High-flow nasal cannula oxygen therapy is effective for moderate hypercapnic respiratory failure as it helps normalize clinical and gas exchange levels with an acceptable rate of nonresponders who require ventilatory support.

## Abstract (pt)

**Objetivo:**

Avaliar a eficácia e a segurança da oxigenoterapia com uso de cânula nasal de alto fluxo no tratamento da insuficiência respiratória hipercápnica moderada em pacientes que não conseguem tolerar ou têm contraindicações para ventilação mecânica não invasiva.

**Métodos:**

Estudo prospectivo observacional de 13 meses envolvendo participantes admitidos a uma unidade de terapia intensiva com insuficiência respiratória hipercápnica ou durante o processo de seu desenvolvimento. Os parâmetros clínicos e de troca gasosa foram registrados em intervalos regulares durante as primeiras 24 horas. Os parâmetros finais foram saturação de oxigênio entre 88 e 92%, juntamente da redução do esforço respiratório (frequência respiratória) e da normalização do pH (≥ 7,35). Os participantes foram considerados não responsivos em caso de necessidade de utilização de suporte ventilatório.

**Resultados:**

Trinta participantes foram tratados utilizando oxigenoterapia com cânula nasal de alto fluxo. Esta foi uma população mista com exacerbação de doença pulmonar obstrutiva crônica, edema pulmonar cardiogênico agudo, e insuficiência respiratória aguda pós-operatória e pós-extubação. Observou-se melhora não significante na frequência respiratória (28,0 ± 0,9 *versus* 24,3 ± 1,5; p = 0,22), que foi aparente nas primeiras 4 horas do tratamento. Ocorreu melhora do pH, embora só se tenham obtido níveis normais após 24 horas de tratamento com cânula nasal de alto fluxo (7,28 ± 0,02 *versus* 7,37 ± 0,01; p = 0,02). A proporção de não responsivos foi de 13,3% (quatro participantes), dos quais um necessitou e aceitou ventilação mecânica não invasiva, e três necessitaram de intubação. A mortalidade na unidade de terapia intensiva foi de 3,3% (um participante), e um paciente morreu após a alta para a enfermaria (mortalidade hospitalar de 6,6%).

**Conclusão:**

O oxigenoterapia com cânula nasal de alto fluxo é eficaz para a insuficiência respiratória hipercápnica moderada e ajuda a normalizar os parâmetros clínicos e de troca gasosa, com taxa aceitável de não responsivos que necessitaram de suporte ventilatório.

## INTRODUCTION

The first line of treatment for acute hypercapnic respiratory failure - apart from measures for controlling causative and precipitating factors - is oxygen therapy.^(1)^ The purpose of this treatment is to prevent the development of hypoxemia and the resulting tissue hypoxia. However, oxygen should be administered in a strictly controlled way to prevent its known adverse effects.^(2)^ Complications of oxygen therapy include hypercapnia and acidosis caused by several pathophysiological mechanisms (Haldane and Bohr effects, inhibition of the respiratory drive), which may result in the patient requiring ventilatory support.^(3)^

In these situations, scientific societies recommend administering a specific oxygen concentration via a high-flow oxygenation system such as Venturi masks.^(4)^ Oxygen saturation (SpO_2_) must be continuously monitored by pulse oximetry and maintained within a narrow interval, ranging between 88 and 92%.^(5)^ In case of hypercapnic acidosis, excessive respiratory work or hypoxemia despite the administration of oxygen > 40%, noninvasive mechanical ventilation (NIV) should be used, unless it cannot be tolerated, is contraindicated or if the patient needs to be intubated.^(6)^

In recent years, high-flow nasal cannulas, the so-called HFNC therapies, have gained popularity, since they deliver high flows (up to 50 - 60L/minute) and accurate concentrations (21 - 100%) of oxygen.^(7,8)^ Furthermore, HFNC can be used in combination with heaters/humidifiers of inspired gas and prongs, which facilitates patient tolerability partly due to the replacement of fitted face masks.^(9)^ High-flow nasal cannulas have been successfully employed in patients with moderate hypoxemic respiratory failure.^(10)^ Some of the mechanisms of action of HFNC can also be effective in the treatment of acute hypercapnic respiratory failure.^(11)^ High-flow nasal cannula provides a washout effect of the upper airway dead space, which reduces hypercapnia. Additionally, it reduces airway resistance and, consequently, the work of breathing. Finally, HFNCs deliver expiratory positive airway pressures (continuous positive airway pressure - CPAP effect) that can counterbalance intrinsic positive end-expiratory pressure (PEEP), which is present in most of these patients.^(12)^

For these reasons, our hypothesis is that - in hypoxemic acute respiratory failure- HFNC can be used as an alternative therapy to NIV in patients in which the latter use is not possible due to intolerance or is contraindicated.^(13)^ Initially, as with any other therapy that has not been previously tested in robust clinical trials, HFNC could be used for mild to moderate hypercapnic respiratory failure in settings where patient safety is preserved and under the supervision of independent research ethics committees.^(14)^

The purpose of this study was to assess the efficacy and safety of HFNC oxygen therapy in the treatment of moderate hypercapnic respiratory failure as an alternative to NIV in the context of a treatment protocol in patients of an intensive care unit (ICU).

## METHODS

This is a prospective, observational study conducted between October 1, 2014 and November 30, 2015 involving ICU patients with moderate acute hypercapnic respiratory failure who received HFNC therapy as part of a treatment protocol established for this condition. The study was approved by the Ethics Committee of the hospital that waived the need for consent for reviewing medical records. All data were disaggregated, anonymized and entered into a database.

**Figure 1:**
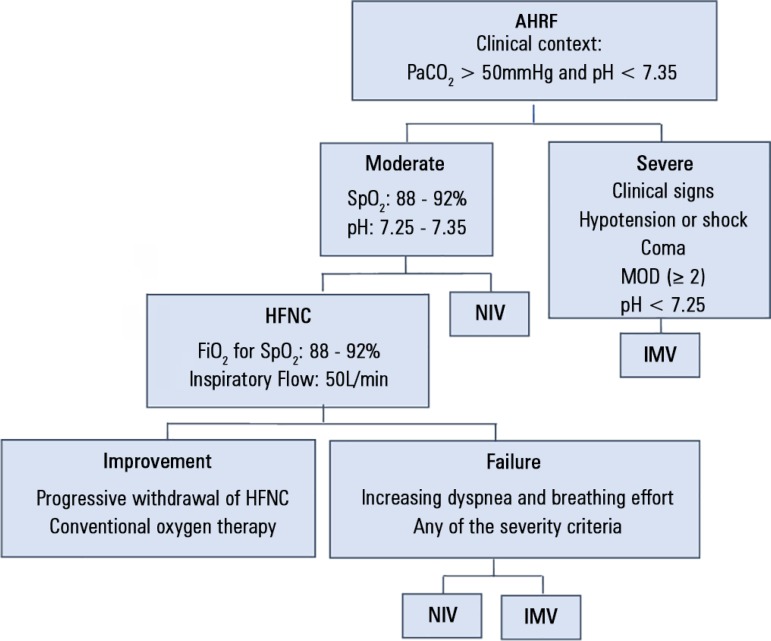
Protocol for the treatment of hypercapnic acute respiratory failure. AHRF - acute hypercapnic respiratory failure; PaCO_2_ - partial pressure of carbon dioxide; SpO_2_ - oxygen saturation; MOD - multiple organ dysfunction; HFNC - high-flow nasal cannula; FiO_2_ - inspired fraction of oxygen; NIV - noninvasive ventilation; IMV - invasive mechanical ventilation.

All patients admitted to the ICU with a diagnosis of hypercapnic acute respiratory failure episode were treated following a treatment protocol previously implemented in clinical practice, which included the following devices: (1) nasal cannula set at a flow of up to 5L/min; (2) Venturi face mask set up to 40%; (3) HFNC oxygen therapy; (4) NIV; and (5) invasive mechanical ventilation (IMV). The treatment was initiated with one of these devices according to the clinical status of the patient, then depending on the patient's responsiveness and course of disease, the therapy was maintained or shifted to another device. Complementarily, patients received the conventional therapies indicated for their status according to standard clinical practice guidelines (bronchodilators, corticosteroids, antibiotics, etc.). The diagnostic criteria for hypercapnic acute respiratory failure were carbon dioxide venous pressure (p_v_CO_2_) > 50mmHg and pH < 7.35 in a compatible clinical context. The criteria for initiating HFNC oxygen therapy in patients with respiratory failure were persistent SpO_2_ < 88%, despite the use of an oxygen face mask with fraction of inspired oxygen (FiO_2_) set at ≥ 40%. Eligible patients were those who could not tolerate the interface or had relative or absolute contraindications to NIV. At this point, the choice of HFNC therapy over NIV, IMV or continuing oxygen face mask was at the physician's discretion. The exclusion criteria were as follows: pH ≤ 7.25; Glasgow coma scale < 12 points; dysfunction of multiple organs > 2, respiratory included; and clinical and metabolic criteria for shock. This means that patients were excluded if they required immediate invasive (or noninvasive) mechanical ventilation (MV). The criteria for discontinuation of HFNC therapy were a) patient improvement with stable SpO_2_ ≥ 88%, FiO_2_ ≤ 0.4 with a flow below 25L/min; and b) worsening of the patient's condition due to intolerance to treatment, persistent or worsening dyspnea, persistent abdominal paradox, respiratory rate ≥ 35rpm, systolic blood pressure < 90mmHg, SpO_2_ < 88%, increase in p_v_CO_2_ by > 10mmHg and/or decrease in pH by > 0.08; and c) patient refusal. Figure 1 shows a setup of the protocol implemented.

Data monitoring and collection were performed at specific intervals, as follows: (t0) or baseline time, at patient's admission to the ICU with their previous oxygen therapy; (t1) 1 - 4 hours after initiation of HFNC therapy; (t2) 5 to 8 hours; (t3) 9 to 12 hours; and (t4) 13 to 24 hours. The null hypothesis was that no significant differences would be observed between the study periods concerning the study variables. This was tested using ANOVA for repeated measures and a post hoc test (Scheffé's test) between them when statistically significant differences were observed. A p < 0,05 was considered significant. The statistical software employed was Statistical Package for Social Science (SPSS), version 15.

Follow-up of patients was performed from patient admission to the ICU until hospital discharge. Other variables included demographics (age and sex); etiology of the respiratory failure; level of severity as estimated by Acute Physiology and Chronic Health Evaluation II (APACHE II); time on HFNC; need for NIV or endotracheal intubation; stay in the ICU; and mortality (in ICU and in hospital). Patients who received palliative HFNC therapy were excluded from the analysis.

**Figure 2:**
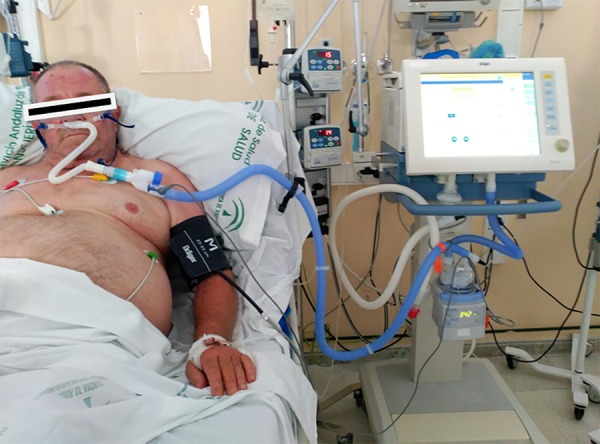
Set up of high-flow nasal cannula oxygen therapy.

High-flow nasal cannula was administered via an Evita-XL de Dräger^®^ ventilator set at "oxygen therapy" mode, which constantly controls FiO_2_ (0.21 - 1) and flow (2L/min at 50L/min). Optimal gas conditioning was achieved by the use of a MR850 heated humidifier with an RT340 dual-heated breathing circuit connected in series to a set of OptiFlow(tm) nasal cannulas (Fisher & Paykel) (Figure 2).

## RESULTS

**Figure 3:**
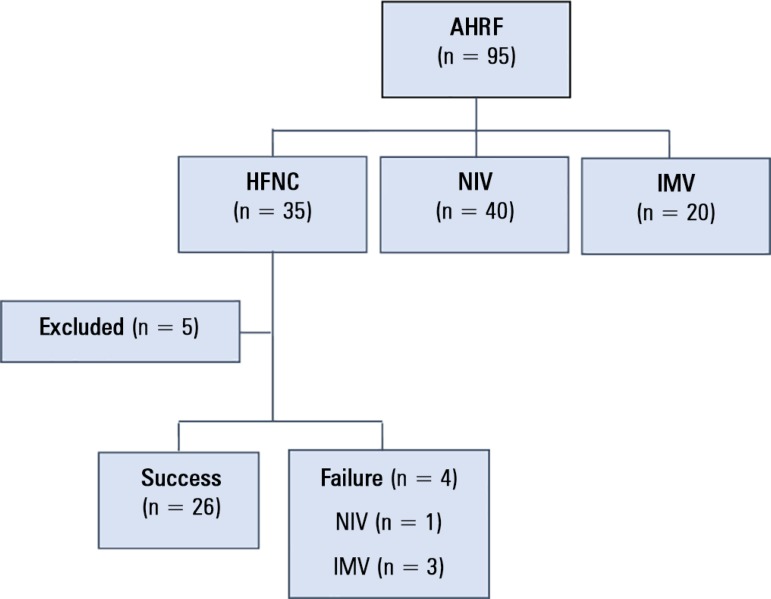
Patient flowchart. AHRF - acute hypercapnic respiratory failure; HFNC - high-flow nasal cannula; NIV - noninvasive ventilation; IMV - invasive mechanical ventilation.

Of the 1,304 patients admitted to the ICU during the study period, 35 received HFNC therapy for acute or chronic respiratory failure. High-flow nasal cannula oxygen therapy was palliative in five of the 35 patients and was excluded from the study. A patient flowchart is shown in figure 3. The mean age for the patients in this group was 66.7 years ± 12.9 (95%CI 65.3 - 70.1), of whom 20 (66.6%) were men. The mean APACHE II was 16.9 ± 6.5 (95%CI 15.7 - 18.1).

With regard to the etiologies of respiratory failure, the most common were *chronic obstructive pulmonary disease* (COPD): 20 (66.6%); congestive heart failure: 5 (16.6%); and sleep-related or obesity hypoventilation: 5 subjects (16.6%). Ten episodes were at admission to the ICU and 20 after extubation from the IMV (postoperative, trauma and others). The reasons for not using NIV as the first choice were as follows: 12 not tolerant to the oronasal mask (anxiety and uncooperative); 10 copious secretions or inability to cough; 3 esophageal or gastric surgery; 2 mismatch between mask and face and not stated in 3.

The clinical effects of HFNC included a reduction in respiratory rate (although it was not statistically significant) (28.0 ± 0.9 *versus* 24.3 ± 1.5, p = 0.22) within four hours after initiation of the HFNC therapy.

The effects of HFNC on gas exchange parameters are shown in table 1. Of note is the significant improvement observed in pH, although normal levels were only reached after 24 hours following the initiation of the HFNC therapy (0.02 *versus* 7.37 ± 0.01, p = 0.02). Post hoc analysis revealed statistically significant differences in pH between baseline and all the other times, as well as between t1 and t4.

**Table 1:** Clinical and gas exchange parameters

	t0	t1	t2	t3	t4	p value
RR (rpm)	28.0 ± 0.9 (26.0 - 29.9)	25.9 ± 1.1 (23.4 - 28.6)	25.6 ± 1.3 (22.8 - 28.4)	24.7 ± 1.4 (21.4 - 27.9)	24.3 ± 1.5 (20.7 - 27.9)	0.22
SpO_2_	89.7 ± 1.3 (87.9 - 92.5)	92.6 ± 0.8 (91.0 - 94.2)	91.7 ± 1.3 (88.8 - 94.5)	91.2 ± 1.6 (87.7 - 94.7)	91.1 ± 0.7 (89.7 - 92.5)	0.58
SpO_2_/FiO_2_	228.5 ± 19.3 (189.0 - 267.9)	184.9 ± 11.2 (133.1 - 167.3)	213.4 ± 14.3 (183.6 - 243.1)	212.5 ± 13.3 (184.3 - 240.7)	230.5 ± 17.7 (191.5 - 269.6)	0.23
PvCO_2_ (mmHg)	72.3 ± 4.0 (62.7 - 81.9)	69.3 ± 4.5 (59.9 - 78,78)	67.5 ± 5.18 (56.7 - 78.2)	66.7 ± 6.7 (52.3 - 81.1)	58.0 ± 4.7 (47.6 - 68.4)	0.59
pH	7,28 ± 0.02* (7.25 - 7.32)	7.31 ± 0.02† (7.27 - 7.34)	7.32 ± 0.02 (7.28 - 7.37)	7.34 ± 0.02 (7.29 - 7.38)	7.37 ± 0.01 (7.35 - 7.40)	0.02
HCO_3_ (mmol/L)	32.3 ± 1.2 (29.9 - 34.8)	34.2 ± 1.6 (30.8 - 37.6)	33.9 ± 1.9 (29.8 - 37.9)	35.3 ± 2.5 (29.9 - 40.8)	33.7 ± 1.9 (29.4 - 38.0)	0.75

RR - respiratory rate; SpO_2_ - oxygen saturation by pulse oximeter; FiO_2_ - inspired fraction of oxygen; pvCO_2_ - carbon dioxide venous pressure; HCO_3_ - bicarbonate plasma concentration.

* RR - respiratory rate; SpO_2_ - oxygen saturation by pulse oximeter; FiO_2_ - inspired fraction of oxygen; pvCO_2_ - carbon dioxide venous pressure; HCO_3_ - bicarbonate plasma concentration.

† t1 *versus* t4. Results expressed as mean ± standard deviation (interquartile range.

Of the 30 patients who received HFNC, four subjects (13.3%) required MV, of whom three (10%) required IMV and one (3.3%) required and accepted NIV. The mean stay was 7.3 ± 11.9 days (95%CI 5.1 - 9.5) in the ICU and 15.5 ± 12.8 days (95%CI 13.2 - 17.9) in the hospital. One patient in this group died in the ICU (3.3%), and another subject died in the hospital, which represents a mortality rate of 6.6%.

## DISCUSSION

The data obtained demonstrate that HFNC is clinically effective, since it reduces respiratory rate and reverses respiratory acidosis. The protocol implemented in our ICU in moderate cases, introducing HFNC as an alternative to NIV when there is intolerance to the interface or relative/absolute contraindications to its use, has been shown to be safe in this setting, with an acceptable rate of ventilatory support rescue.

The use of HFNC was associated with a reduction in respiratory rates, although it had no effect on p_v_CO_2_, which is consistent with the results reported in previous clinical and pathophysiological studies.^(10,14-16)^ The fact that a decrease in respiratory frequency was not accompanied by an increase in p_v_CO_2_ is due to the HFNC washout effect of the upper airway dead space. Conversely, a statistically significant increase was observed in pH, probably caused by the slight decrease in p_v_CO_2_ which, although it was not significant, had an effect on the acid-base status. Interestingly, pH values within the first hours after the initiation of the treatment increased from 7.28 ± 0.18 to 7.31 ± 0.18, which are very similar to the increase from 7.27 ± 0.10 to 7.31 ± 0.09 reported by Brochard on his seminal study on NIV.^(17)^ Furthermore, although respiratory work and oxygen cost were not measured, they must have presumably been reduced as a result of the improvement achieved on ventilatory efficiency. The high flow rates employed produce an expiratory pharyngeal pressure and hence a CPAP effect, which can counterbalance the intrinsic PEEP and airway inspiratory resistance present in these patients.

The clinical outcomes obtained with the use of HFNC for decompensated chronic respiratory failure are consistent with the moderate severity of the patients who received this therapy, with lower intubation and mortality rates than that reported for patients with NIV.^(18)^ Thus, our rate of intubation was 10% for HFNC (18 - 28% reported for NIV), and the hospital mortality rate was 6.6% (*versus* 10 - 13% for NIV).

The use of HFNC in adult patients with acute on chronic respiratory failure is still anecdotical, and only a few case-report studies have been published. Thus, Millar et al.^(19)^ reported the use of HFNC for the management of a patient with chronic hypercapnic respiratory failure who did not tolerate NIV. Patient tolerability was achieved due to improvement in patient comfort and reversion of pathophysiological alterations. Similarly, Díaz-Lobato et al.^(20)^ reported the case of a patient with amyotrophic lateral sclerosis who presented in the emergency department with acute hypercapnic respiratory failure. Since the patient did not tolerate NIV and refused intubation, she was successfully treated with HFNC as evidenced by the improvement in pH and partial pressure of carbon dioxide (PaCO_2_) achieved, regaining consciousness and being discharged after five days of hospitalization. The authors stated that her response to HFNC was similar to that expected for NIV. The only case-series study where patients with COPD were not excluded was that conducted by Rittayamai et al.,^(21)^ where the etiology of respiratory failure was an exacerbation of COPD in 6 of the 17 patients. Although these patients were not analyzed separately and the use of HFNC was immediately after extubation (as a preemptive therapy), the effects obtained were similar to those observed in our study. Recently, a retrospective analysis of 33 patients in a medical intensive care unit with acute hypercapnic respiratory failure and the use of HFNC was published.^(22)^ Thus, patients and settings were similar to ours but probably they had less severe disease because the pH was in the normal range. They found a slight decrease in PaCO_2_ (approximately 4mmHg in the first hour), similar to our results. In a more complete physiological study on the effect of HFNC on neuroventilatory drive and work of breathing of 14 patients with hypercapnic failure in the postextubation period, Di Mussi et al.^(23)^ did not find any differences in the breathing pattern and gas exchange compared to those observed with oxygen delivered through a face mask. Again, patients had a pH in the normal range, indicating a less severe condition.

Regarding stable patients, a range of studies on the effects of HFNC on ventilatory parameters support the findings of our study. Bräunlich et al. evaluated the effect of HFNC in healthy volunteers, COPD patients, and idiopathic pulmonary fibrosis patients.^(24)^ Compared with unaided breathing, V_T_ increased in patients in the COPD and idiopathic pulmonary fibrosis groups, while it decreased in healthy volunteers. The respiratory rate and minute volume decreased in all groups. Nilius et al. investigated the effects of HFNC in COPD patients with chronic hypercapnic respiratory failure;^(25)^ although there was a high interindividual response, in general terms, respiratory rates and PaCO_2_ were reduced. Chatila et al.^(26)^ observed increased exercise capacity with improved oxygenation via HFNC compared to spontaneous breathing in patients with COPD in an unloaded bicycle ergometer test. Okuda et al. used HFNC to improve sleep-related hypoventilation in a patient with COPD.^(27)^ In conclusion, there is emerging evidence that HFNC is a highly promising treatment for some types of hypercapnic respiratory failure. Two protocols for such studies have been recently published.^(28,29)^

According to the available evidence, one of the most remarkable aspects of HFNC is the high patient acceptability and comfort observed, since this system allows patients to eat, drink, talk, cough and clear secretions. In 2010, Masclans et al.^(30)^ investigated the effects of a HFNC system on dyspnea, mouth dryness and overall comfort as measured by a visual analog scale versus conventional Venturi^®^ masks in the treatment of 20 ICU patients with acute respiratory failure. The HFNC was associated with less dyspnea and mouth dryness and was found to be more comfortable than face masks. Schwabbauer et al.^(31)^ compared the subjective degree of dyspnea (according to the Borg scale), the general level of discomfort and a general evaluation of each type of therapy (Venturi mask *versus* HFNC *versus* NIV). The scores were higher for HFNC in all dimensions than for NIV. The main limitation of both studies is the short period of observation, which was less than one hour. In our study -where the time on HFNC therapy and follow-up period were longer- and in agreement with the studies by Carratalá Perales et al.^(32)^ and Tiruvoipati,^(33)^ no remarkable adverse effects were observed, and only one patient rejected the HFNC system due to discomfort.

The main limitation of this study is its observational design, which may involve a selection bias. As this is a noncontrolled, pretest/posttest study, it allows us to assess the efficacy of a measure, but we cannot be certain that the improvements observed were due to the intervention. The admission policy of our ICU involves the admission of patients who can be potentially cured and the rejection of those with advanced illness and a very poor prognosis, although their physiological respiratory parameters during decompensation may be similar. Although the quantitative criteria for the initiation of HFNC therapy are clearly established in the protocol, the clinical judgment of the severity of symptoms has a subjective component that may influence the type and timing of therapy initiation. Nonetheless, as the clinical judgment of physicians has been demonstrated to be better correlated with the course and prognosis of disease, it must be taken into account in all protocols.^(34)^ Another limitation of this study is that venous gases instead of arterial gases were considered for patient control and follow-up, together with the SpO_2_ and the SpO_2_/F_i_O_2_ ratio. This choice was made to prevent complications from repeated arterial punctures and/or cannulation when hemodynamic control was not indicated, as well as to reduce the use of invasive methods and avoid work overload for the nursing staff. It has been demonstrated that a correlation and concordance exist between pH and bicarbonate values in venous and arterial gasometry^(35)^ when no variations occur in cardiac output and carbon dioxide production.^(36)^ Thus, in our study, a good pH/bicarbonate concordance can be assumed, since patients with shock, hypotension, increased lactic acid levels or who were in a hypermetabolic state were excluded.

Finally, in accordance with the existing literature, we advise against the indiscriminate use of HFNC.^(37-39)^ The easy administration and follow-up of HFNC therapy may provide a false sense of safety. However, this type of therapy should be administered to selected patients, and follow-up should be performed by trained personnel who can frequently assess treatment response and perform immediate intubation and MV when necessary.

## CONCLUSION

This preliminary study demonstrates that high-flow nasal cannula therapy is effective in improving clinical and gas exchange parameters in patients with moderate hypercapnic respiratory failure, with an acceptable rate in nonresponders who required ventilatory support. These results should be confirmed with rigorous clinical trials before being translated into clinical practice.
